# JAGuaR: Junction Alignments to Genome for RNA-Seq Reads

**DOI:** 10.1371/journal.pone.0102398

**Published:** 2014-07-25

**Authors:** Yaron S. Butterfield, Maayan Kreitzman, Nina Thiessen, Richard D. Corbett, Yisu Li, Johnson Pang, Yussanne P. Ma, Steven J. M. Jones, İnanç Birol

**Affiliations:** Canada's Michael Smith Genome Sciences Centre, Vancouver, BC, Canada; INRA Clermont-Ferrand Research Center, France

## Abstract

JAGuaR is an alignment protocol for RNA-seq reads that uses an extended reference to increase alignment sensitivity. It uses BWA to align reads to the genome and reference transcript models (including annotated exon-exon junctions) specifically allowing for the possibility of a single read spanning multiple exons. Reads aligned to the transcript models are then re-mapped on to genomic coordinates, transforming alignments that span multiple exons into large-gapped alignments on the genome. While JAGuaR does not detect novel junctions, we demonstrate how JAGuaR generates fast and accurate transcriptome alignments, which allows for both sensitive and specific SNV calling.

## Introduction

Deep sequencing of transcriptomes on high throughput sequencing platforms, also called RNA-seq, is an effective technique for interrogating transcript expressions. The data type also provides nucleotide level sequence information, allowing for variant detection, alternative splicing, and novel transcript discovery, among other uses. In cancer studies variant detection from RNA-seq data is important for identifying potential driver mutations for disease, so there is a need for good quality RNA alignment tools that support sensitive and accurate variant calling.

However, with increasing read length, read sequences can often span one or more exon-exon junctions, making it challenging to align them to genomic sequence alone. A number of tools have been developed to address this. TopHat and TopHat2 [1], [2] use a Burrows Wheeler Transform [3] to align reads to the reference genome, followed by alignment of the remaining reads to splice sites identified on the reference genome. GSNAP [4] detects read splicing using probabilistic models or a database of known splice sites. MapSplice [5] first splits reads into segments, and maps them to a reference genome by using Bowtie [6]. It then attempts to map remaining unmapped segments as gapped alignments, with each gap corresponding to a splice junction. Tools can subsequently be used to find intra-chromosomal read pairs left unaligned by previous stages [7]. SpliceMap splits reads, aligns them, and the half-reads are then pieced together to determine locations of exons and junctions [8]. TrueSight takes all possible splice junctions of one transcriptome and uses a regression model to find the best assignment for them [9]. OLego adopts a multiple-seed-and-extend scheme for de novo spliced mapping of mRNA-seq reads, and does not rely on a separate external mapper [10]. Another complementary approach aimed at improving the sensitivity of RNA-seq alignment in the presence of variation is based on hash table representations of the genome [4]. STAR aligns RNA-seq reads to a reference genome using uncompressed suffix arrays [11]. PASTA first aligns reads to the genome and then splits unaligned reads across junction regions [12]. SOAP is useful for detecting the junctions for those mRNAs with relatively lower expression levels [13].

Most methods combine the alignment of gapped and un-gapped reads, requiring the use of their own particular alignment algorithm, and do not work with different aligners. BWA [14] is a well-established alignment algorithm that is used extensively for high throughput analysis and has been cited in over 500 bioinformatics publications. JAGuaR offers an annotation-based solution to the RNA-seq alignment problem, and is compatible with pipelines running BWA (here, reported on version 0.5.7 and 0.7.4). JAGuaR uses annotated exon-exon junctions to extend a genomic reference, which is used as a reference. After alignment to this reference, JAGuaR converts reads that align to the exon-exon junction spanning sequences, allowing for large-gapped alignments in genomic coordinates. JAGuaR provides a fast and reliable annotation-based alignment of RNA-seq libraries, which are well-suited to high-throughput clinical and research environments.

## Methods

### The JAGuaR algorithm

JAGuaR first uses a modified GTF (Gene Transfer Format) of known splice sites to build the junction reference sequence (Table S1 in File S1, Figure S2 in File S1). Exon junction spanning sequences are concatenated onto the end of each chromosome in the genome reference to form the JAGuaR reference, which is used as the target sequence for BWA read alignments. This needs to be run once for each size of sequence reads that will be aligned (Figure S1 in File S1, Figure S2 in File S1). The size of sequences flanking exon-exon junctions that are added to the extended reference is dependent on the size of the reads that will be aligned, in order to minimize the number of unspliced reads that align to the junction portion of the extended reference. After reads are aligned to this reference with BWA resulting in a SAM file [15], JAGuaR is used to translate the coordinates of the exon junction aligned reads to genome coordinates providing modified CIGAR (Compact Idiosyncratic Gapped Alignment Report) strings, read pair assessment (FLAG), and mapping qualities.

### Test Datasets

To demonstrate the performance of JAGuaR, three sets of cell line libraries were analyzed: Universal Human Reference RNA from Agilent Technologies (Sample 1 and 2), and HelaS3 (Sample 3). On the Illumina HiSeq 2000 platform, 100 bp paired end reads were sequenced (http://trace.ncbi.nlm.nih.gov/Traces/sra/sra.cgi?study=SRP041367 ^)^. The reference for the alignments in all cases was based on GRCh37-lite (hg19) with corresponding transcript models from Ensembl61 [16], and the UCSC GenomeBrowser [17]. All tools used the same database of known splices sites (the gene annotation file used is available in GTF format on the JAGuaR download site).

In addition to these real datasets, we generated a simulated RNA-seq dataset (Sample 4) using the Flux Simulator software [18] (Text S1 in File S1). In order to simulate allelic expression of SNPs, which were used for evaluation purposes (see Comparisons, below), we ran Flux Simulator twice on reference genomes that we “implanted” with known single nucleotide variant (SNVs). To this end, we called SNVs from the Illumina Body Map 16 tissue mixture library (http://www.ebi.ac.uk/arrayexpress/experiments/E-MTAB-513/). These were separated into two VCFs (Variant Call Format), for variants estimated as homozygous, and heterozygous, respectively. These were each implanted separately into the hg19 reference (GRCh37-lite) using the GATK tool FastaAlternativeReferenceMaker [19] to create two haplotype references. FluxSimulator was run on each reference to produce 100 million paired-end, strand-specific 100-bp reads (see supplementary information for full run parameters). Finally, the fastq files produced by the two haplotype simulations were renamed, merged, filtered for reads <100 bp long, and split into read1 and read2 fastq files for subsequent alignment and analysis.

### Comparisons

We compared the performance of JAGuaR (v2.1) with three other popular split read alignment tools, TopHat2 (v2.0.8b), GSNAP (v2012-12-12) and MapSplice (v2.1.5). We also attempted to compare to SpliceMap [8], TrueSight [9] and OLego [10]. In our software comparison we required that a tool be successfully installed and running within 3 days of active effort to allow for operating system dependencies and communication with developers. Under this criteria TrueSight and OLego were eliminated due to repeated segmentation faults and SpliceMap due to problems in loading Bowtie. All issues were communicated with the developers but were not resolved within the testing timeframe. We also compared the performance of JAGuaR using BWA (v0.5.7) and BWA-MEM (v0.7.4). As BWA-MEM is able to align reads that are split across more than one genomic location, we also included a comparison of JAGuaR used in conjunction with BWA-MEM to running BWA-MEM only. The split alignments are reported as secondary aligments in BWA-MEM and for the purposes of the comparison we only chose the alignment which aligned the most bases, which results in a slight undercount for the number of junction spanning reads detected by BWA-MEM alone.

JAGuaR was run with BWA at default settings where -t (number of threads) is set to 1. TopHat2, was run with -p (number of threads) set to 4, "—no-novel-juncs" set, and the GTF annotation file specified. MapSplice was run with -p (number of threads) set to 4. GNSAP was run with -B (batch mode) set to 5, -t (number of worker threads) set to 8 and the specified GTF annotation file (converted to binary format). As GSNAP ran much slower than the other tools, we increased the number of threads used so the analysis would complete in a reasonable amount of time. The output which includes multiple alignments for a read was filtered for the first two paired ends of the highest quality. This was also done for JAGuaR/BWA-MEM.

We compared the performance of the methods on the simulated dataset as well as the cell-line samples. In the absence of ‘truth’ data for the cell-line samples, we used the total reads aligned and the number of unique exon-exon junctions that were covered by at least one read as metrics to estimate alignment accuracy and sensitivity of each tool. In addition, dbSNP [20] concordance can also be used as a measure of sensitivity and specificity of RNA-seq alignment, and RNA-seq SNP data is important in disease models where the associated gene is expressed. Therefore, we further evaluated the tools by comparing annotated single nucleotide variant (SNV) calls (Samtools v0.1.12a, mpileup [15], snpEff [21] and snpSift [22]) with common variants tracked in the dbSNP v137 (NCBI) (minor allele frequency, MAF > = 0.01).

### Ethics Statement

The samples used are derived from commercially available cell lines. The work described herein was conducted at the BC Cancer Agency's Michael Smith Genome Sciences Centre, reviewed by the University of British Columbia - BC Cancer Agency Research Ethics Board.

## Results and Discussion

**Figure 1:**
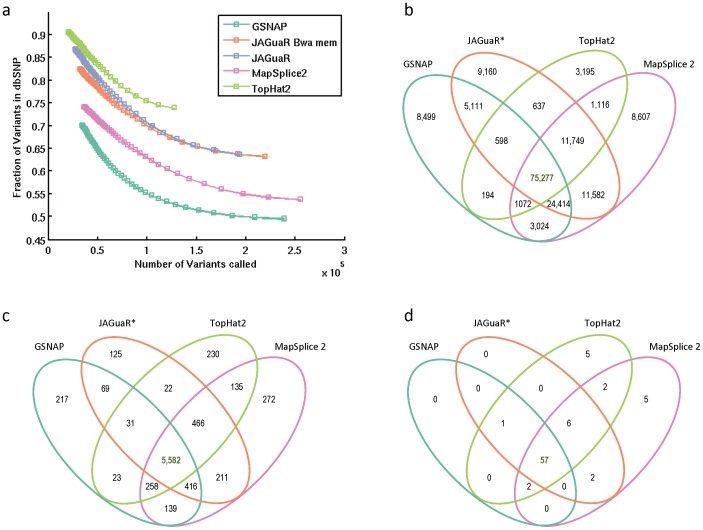
SNV concordance between tools for one read set (Sample 2). a) Number of variants in dbSNP (v137) plotted against number of variants called at various levels of depth. Depth begins on far right at 6 bp and each point represents increasing depth of 1 bp coverage. b) Overlap of known SNVs called c) Overlap of known non-synonymous SNVs called d) Overlap of SNVs called in COSMIC. All SNP calls were assessed at depth of 6. *BWA-MEM.

JAGuaR performed well when comparing the number of identified exon-exon junctions, sequence coverage of junctions, and the number of dbSNP concordant SNVs called (Table 1). Despite the similarity in alignment metrics, the number of SNVs called is very different between the methods. JAGuaR calls show improved sensitivity compared to TopHat2 due to higher number of concordant SNVs and higher specificity compared to GSNAP and MapSplice2 due to a higher concordance of calls with dbSNP (v137)(Table 1). SNV comparisons are based on a minumum coverage of 6 reads in order to maximize the number of SNVs used for comparison while still maintaining dbSNP concordance of >50% for all tools. The rank of all tools by dbSNP concordance remains the same at all depths (Figure 1a). The increased sensitivity over TopHat2 and specificity over the other two tools is further seen when the total number of dbSNP concordant calls are plotted against the fraction of dbSNP concordance in each sample (Figure S3a in File S1, Figure S4a in File S1).

**Table 1:** Alignment results of three paired end tag (PET) RNA-seq libraries.

Read set/Tool	Reads	Aligned	Junction aligned reads	Junctions covered (> = 1 read)	SNVs called	Concordant with dbSNP	Fraction Concordant
Sample 1							
JAGuaR	269,842,308	245,432,525	62,433,477	231,909	357,080	206,249	0.58
JAGuaR*	269,842,308	262,411,159	60,906,549	231,565	364,594	213,743	0.59
GSNAP	269,842,308	264,518,583	67,241,881	229,098	366,912	210,976	0.58
MapSplice2	269,842,308	268,996,985	54,420,016	207,030	437,594	218,544	0.50
TopHat2	269,842,308	255,018,014	68,421,007	246,147	249,223	169,618	0.68
Sample 2							
JAGuaR	145,794,964	105,967,294	23,820,302	215,619	193,565	123,289	0.64
JAGuaR*	145,794,964	129,061,693	25,923,444	217,935	219,346	138,728	0.63
GSNAP	145,794,964	119,860,144	26,361,372	212,238	238,572	118,389	0.50
MapSplice2	145,794,964	124,942,385	22,470,234	186,656	255,261	137,093	0.54
TopHat2	145,794,964	106,051,847	24,450,626	225,873	127,318	94,090	0.74
Sample 3							
JAGuaR	200,670,094	185,914,022	51,501,965	185,284	318,610	186,557	0.59
JAGuaR*	200,670,094	195,801,212	50,234,507	185,569	327,985	193,013	0.59
GSNAP	200,670,094	197,561,649	55,491,975	183,501	316,826	179,954	0.57
MapSplice2	200,670,094	200,608,733	46,228,494	164,294	385,229	192,750	0.50
TopHat2	200,670,094	197,698,328	56,336,845	198,157	238,883	160,635	0.67

*BWA-MEM algorithm.

Comparison of alignment statistics and SNP concordance with dbSNP (v137) between JAGuaR, GSNAP, MapSplice2 and TopHat2. SNVs were identified with a minimum total coverage of 6 reads in order to maximize the number of SNVs for comparison while maintaining a minimum dbSNP concordance of 50% for all tools. See Figure 1 for ROC plot of Sample 2 (Figure S3 for Sample 1 and Figure S4 for Sample 3).

The overlap between SNVs called using JAGuaR (with BWA and BWA-MEM), GSNAP, MapSplice2 and TopHat2 from each of the samples was analyzed. This was done for the subset of known SNVs in dbSNP, known non-synonymous SNVs, and those seen in the COSMIC database (Figure 1, Figure S3 in File S1, Figure S4 in File S1).

With further filtering based on non-synonymous SNVs and those in the COSMIC database, concordance between all tools is higher. The number of SNVs called in each category is quite similar, showing that the majority of the SNVs are called by all methods.

Memory usage and the length of time it took each tool to process a set of paired end RNA-seq reads into a BAM formatted alignment file are reported in Table 2. From fastq reads to BAM file, JAGuaR in combination with BWA-MEM gives the fastest runtime out of the methods tested.

**Table 2:** Execution Time.

Read set/Tool	Total Time (fastq to bam)	Hours	Memory (GB)
Sample 1			
JAGuaR	4206	70.10	0.8 (9)**
JAGuaR*	905	15.08	0.8 (9)**
GSNAP	4872	81.20	17.9
MapSplice2	4493	74.88	5.3
TopHat2	2540	42.33	3.5
Sample 2			
JAGuaR	3634	60.57	0.8 (9)**
JAGuaR*	296	4.93	0.8 (9)**
GSNAP	3974	66.23	17.9
MapSplice2	1698	28.30	5.3
TopHat2	1636	27.27	3.5
Sample 3			
JAGuaR	2363	39.38	0.8 (9)**
JAGuaR*	513	8.55	0.8 (9)**
GSNAP	2977	49.62	17.9
MapSplice2	3037	50.62	5.3
TopHat2	1593	26.55	3.5

*BWA-MEM algorithm.

**BWA memory.

Comparison of execution time and memory usage. All tools were run on a node with 64 GB memory with no other applications running.

We also compared the performance of JAGuaR with BWA-MEM alone using sample 1 by examining the coverage at exon boundaries. The combination of JAGuaR with both BWA and BWA-MEM increases the dbSNP concordance of called SNPs. JAGuaR combined with BWA-MEM also calls more known SNVs than BWA-MEM alone (Table S3 in File S1). Further, comparing coverage on exon boundaries, we observed 22% of them have increased coverage of 40% with the addition of JAGuaR to BWA-MEM (Figure S5 in File S1).

In addition, we compared all tools against a simulated RNA-seq dataset generated as described in the Methods section. Table 3 shows the number of SNVs called after alignment by each tool. Recovered SNVs are those that are both expected and called. MapSplice2 produces an alignment that recovers the most SNVs, followed by GSNAP, JAGuaR/BWA-MEM, JAGuaR/BWA, and finally TopHat2. SNVs that were expected but not called were generally in intronic regions or in areas that were not covered by the reads generated in the simulation. In this analysis JAGuaR was not the best but was within 4–5% of the best.

**Table 3:** Simulation.

Filtered expected SNPs	25262				
	JAGuaR	JAGuaR*	GSNAP	MapSplice 2	TopHat2
Number called SNPs	38670	44745	30371	69259	20934
Number recovered SNPs	18694	18907	19199	19789	18097
Ratio (recovered/planted)	0.74	0.75	0.76	0.78	0.72

*BWA-MEM algorithm.

All SNPs called at > = 6 bp depth.

Comparison of SNP calls between tools from a simulated dataset. PET synthetic reads were generated from a reference with 652,256 planted SNPs. These fastqs were aligned to hg19 (hg19+junctions with JAGuaR) and SNPs called. SNPs that were identified by from the alignment of at least one tool and which were in the list of planted SNPs, were considered as the filtered expected SNPs. The number of recovered SNPs are the number of SNPs out of the tool's total set that are seen in the expected list. All tools have a similar ratio. Calls from the MapSplice2 alignment show the highest number of SNPs that were not planted and calls from TopHat2 show the least.

## Conclusion

In summary, by using a genome and exon-exon junction reference model combined with post-alignment analysis, we have created a tool to accurately align paired end transcriptome read sequences of increasing length. JAGuaR is designed to work with a range of read lengths (75 to 300 nucleotides) as provided by modern sequencing platforms. Its computational requirements are comparable to existing methods and fastest when used with BWA-MEM. It offers an improvement in alignment sensitivity over some existing methods while still maintaining a higher specificity over others, as shown by the fact that in all comparisons, SNV calls using JAGuaR alignments provide either a higher dbSNP concordance or a high total number of dbSNP concordant calls over other tools. As variant discovery is an important component of many sequencing projects, as a fast, accurate and sensitive tool JAGuaR offers a valuable functionality to RNA-seq analysis. While JAGuaR is not designed to detect differential gene expression or un-annotated transcripts, novel isoforms may still be reconstructed from JAGuaR-aligned reads provided that such isoforms consist of a new combination of known splice sites. As annotation quality increases in human and other model organisms, an accurate and fast alignment for clinical applications is a priority that JAGuaR satisfies.

## Supporting Information

File S1 **Table S1**, Example Transcript Model. **Figure S1**, JAGuaR first requires the reference genome of interest and a transcript model in order to build the reference of the genome sequence and exon junctions. **Figure S2**, Based on a transcript model (Table S1), JAGuaR assesses each exon-exon junction of all available transcripts. **Figure S3**, SNV concordance between tools for one read set (Sample 1). **Figure S4**, SNV concordance between tools for one read set (Sample 3). **Table S3**, SNV Comparison to running of BWA-MEM alone. **Figure S5**, Comparison of JAGuaR+BWA-MEM/BWA-MEM exon start or stop coverage fraction. **Text S1**, Parameters used for Flux Simulator.
(DOCX)
