# Supplementary material for: JAGuaR: Junction Alignments to Genome for RNA-Seq Reads
Source: PLoS One. 2014 Jul 25;9(7):e102398. doi: 10.1371/journal.pone.0102398 (PMC4111418; doi:10.1371/journal.pone.0102398)
Supplement: File S1 — Table S1, Example Transcript Model. Figure S1, JAGuaR first requires the reference genome of interest and a transcript model in order to build the reference of the genome sequence and exon junctions. Figure S2, Based on a transcript model (Table S1), JAGuaR assesses each exon-exon junction of all available transcripts. Figure S3, SNV concordance between tools for one read set (Sample 1). Figure S4, SNV concordance between tools for one read set (Sample 3). Table S3, SNV Comparison to running of BWA-MEM alone. Figure S5, Comparison of JAGuaR+BWA-MEM/BWA-MEM exon start or stop coverage fraction. Text S1, Parameters used for Flux Simulator. (DOCX) [file pone.0102398.s001.docx]

**Table S1** Example Transcript Model

ENST00000566782 HG991_PATCH 66119285 66119659 1 1

ENST00000566782 HG991_PATCH 66298434 66298819 2 1

ENST00000566782 HG991_PATCH 66314236 66314392 3 1

ENST00000566782 HG991_PATCH 66320895 66321004 4 1

ENST00000566782 HG991_PATCH 66339743 66339847 5 1

ENST00000566782 HG991_PATCH 66341024 66341071 6 1

ENST00000566782 HG991_PATCH 66424056 66424100 7 1

ENST00000566782 HG991_PATCH 66440552 66440621 8 1

ENST00000566782 HG991_PATCH 66447170 66447234 9 1

ENST00000566782 HG991_PATCH 66448221 66448294 10 1

A tab delimited transcript text file is required to create the junction reference. Its format currently must be one line per exon for all transcripts in the form of Transcript ID, Chromosome Name (with or without "chr"), Exon Start Coordinate, Exon End Coordinate, Exon Rank in Transcript, Strand. This can easily be created at http://www.ensembl.org/biomart/ by selecting "Ensembl Genes", the genome dataset of interest, "Attributes" and then "Structures". Then choose in this order: Ensembl Transcript ID, Chromosome Name, Exon Chr Start (bp), Exon Chr End (bp), Exon Rank in Transcript, Strand. Export all results to a TSV (tab separated values) file. A custom model can be generated based on a combination of RefSeq, Ensembl and UCFC known genes or novel exons (available on JAGuaR website).


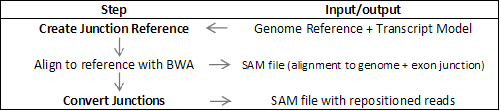

 **Figure S1** JAGuaR first requires the reference genome of interest and a transcript model in order to build the reference of the genome sequence and exon junctions. This step is run once and is required for each length of sequence reads. After reads are aligned to this reference with BWA (Li et. al.) resulting in a SAM file (Li et al. 2009), JAGuaR is used to convert the exon junction aligned reads to genome coordinates. The output file is also in SAM format, and in this file, repositioned reads are annotated with a custom tag. “CIGAR” string summaries of aligned bases are adjusted to offer information on the relative location(s) of the large gap(s) within the read. The BWA algorithm assumes a uni-modal insert size distribution and uses the insert size to assess the status of each read pair; this assumption does not hold for RNA-Seq data, so in addition to repositioning junction-spanning alignments, JAGuaR reassesses whether each read pair is properly mapped using the same approach as BWA, but based on a user-provided maximum insert size. This assessment applies to all reads, not only those that were originally junction aligned. If reads are in a proper pair, their mapping qualities are potentially increased in the same way as was done in the original BWA alignment.

**Figure S2** Based on a transcript model (Table S1), JAGuaR assesses each exon-exon junction of all available transcripts. In this example, for exon 3 and 5, the end of exon 3 and the beginning of exon 5 are concatenated to create ref1. Reads that span this junction are expected to align here. The size of the junction reference is dependent on the length of the reads (L) to be aligned. In order to reduce redundancy in the reference sequence, the size of the portion of exon 3 and 5 use is L-2bp. The same rule applies to exon 4 and 5 to create ref2 as shown. Ref3 and ref4 are examples of cases where L is longer than the size of exon 2. In this case, all of exon 2 is included and L-2 is applied to the adjoining exons. When an alternate transcript exists with exon 2 as in ref4, L-2 applies to the alternate exon 4 while L-2-exon2_length applies to exon 1. The exon junction spanning sequences are then concatenated onto the end of each chromosome in the genome reference to form the JAGuaR reference which will be used as the target sequence for BWA read alignments.

**
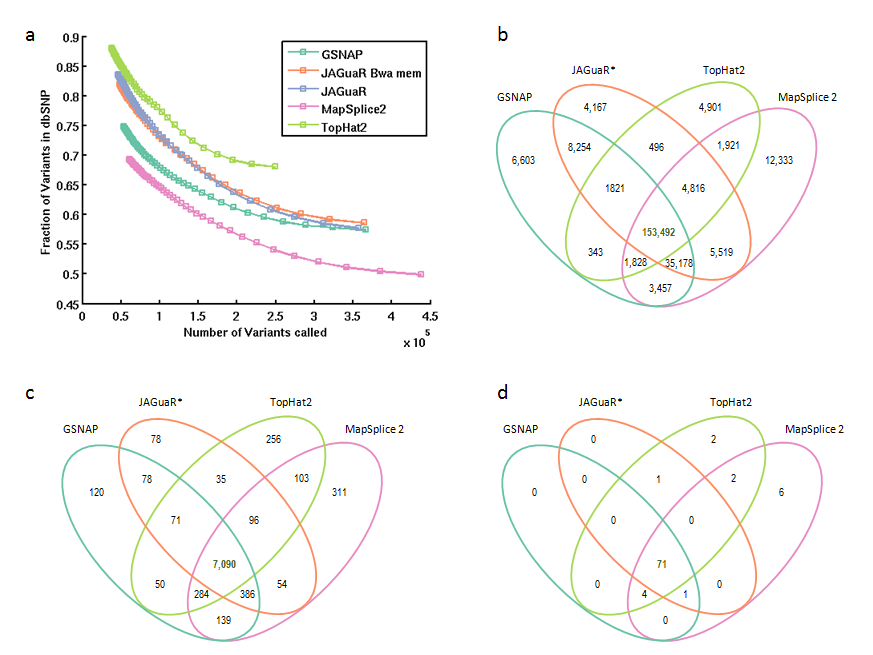
**

**Figure S3** SNV concordance between tools for one read set (Sample 1).

a) Number of variants in dbSNP (v137) plotted against number of variants called at various levels of depth. Depth begins on far right at 6bp and each point represents increasing depth of 1bp coverage.
b) Overlap of known SNVs called

c) Overlap of known non-synonymous SNVs called
d) Overlap of SNVs called in COSMIC. All SNP calls were assessed at depth of 6. *BWA-MEM

**
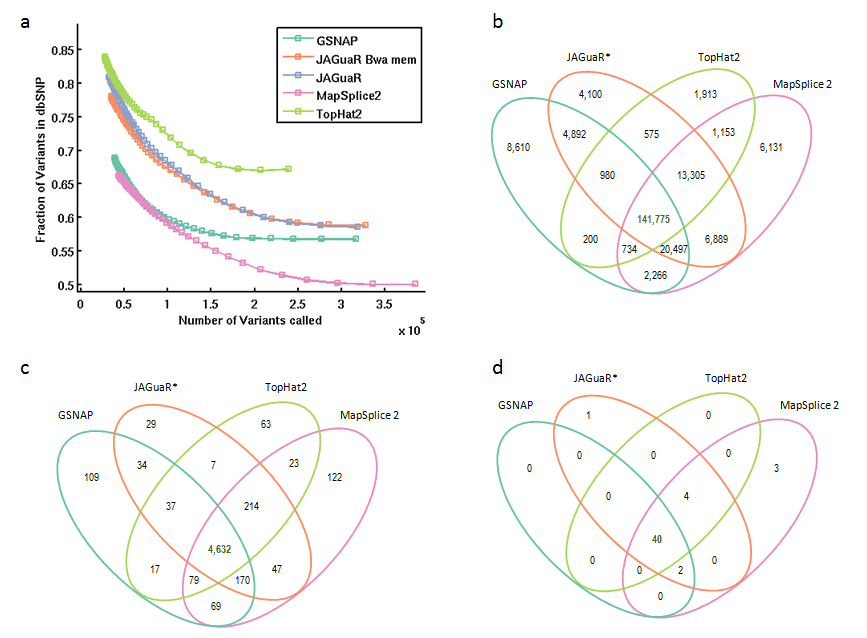
**

**Figure S4** SNV concordance between tools for one read set (Sample 3).

a) Number of variants in dbSNP (v137) plotted against number of variants called at various levels of depth. Depth begins on far right at 6bp and each point represents increasing depth of 1bp coverage.
b) Overlap of known SNVs called

c) Overlap of known non-synonymous SNVs called
d) Overlap of SNVs called in COSMIC. All SNP calls were assessed at depth of 6. *BWA-MEM

**Table S3** SNV Comparison to running of BWA-MEM alone

|  | JAGuaR | JAGuaR* | BWA-MEM only |
| --- | --- | --- | --- |
| Total SNVs | 378600 | 393440 | 445678 |
| Known SNVs | 208560 | 216554 | 216481 |
| dbSNP Concordance | 0.550871 | 0.550411 | 0.485734 |
| *JAGuaR + BWA-MEM |  |  |  |

Running BWA-MEM with JAGuaR repositioning results in a higher SNV concordance (>=6bp coverage) to dbSNP versus BWA-MEM alone. Though running BWA-MEM without a transciptome model, results in more SNVs, a review in IGV shows that many of these are false positive SNVs due to reads that are not split across exon-exon junctions. This results in a lower concordance to dbSNP.


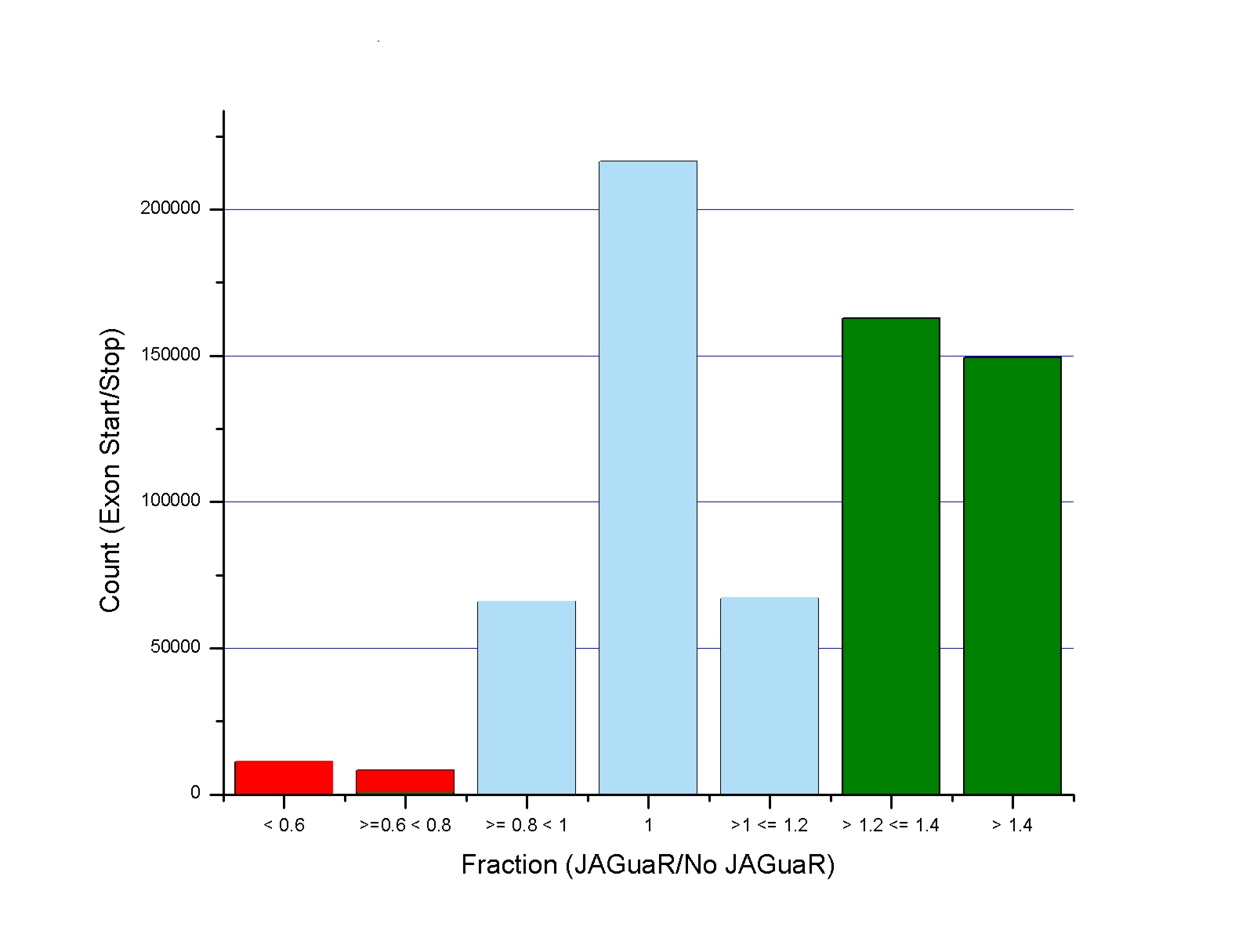


**Figure S5** Comparison of JAGuaR+BWA-MEM/BWA-MEM exon start or stop coverage fraction. Read coverage of all exon boundaries were tabulated from each alignment and the ratio of JAGuaR+BWA-MEM/BWA-MEM alone was plotted. Almost half of exon boundaries have 20% higher coverage with JAGuaR+BWA-MEM (green). Supplementary note 1: Procedure for creating simulated RNA-seq library with bi-allelic SNVs using Flux Simulator.

**Text S1** Parameters used for Flux Simulator

#sort your vcf file to match the headers in the reference genome

perl vcfsorter.pl Homo_sapiens_assembly19.dict Illumina_body_map/HCT20170/ERS025093_5_lanes_dupsFlagged.vcf > Illumina_body_map/HCT20170/ERS025093_5_lanes_dupsFlagged.sorted.vcf 2> Illumina_body_map/HCT20170/vcf_sort.err

# if you want to make a different reference for each haplotype, split the vcf into variants that are hetero- and homo- zygous

# make sure the PL field of the vcf != -1 (this causes GATK to crash)

perl vcf_splitter.pl Illumina_body_map/HCT20170/ERS025093_5_lanes_dupsFlagged.sorted.vcf Illumina_body_map/HCT20170/ERS025093_5_lanes_dupsFlagged.sorted.homozygous.vcf Illumina_body_map/HCT20170/ERS025093_5_lanes_dupsFlagged.sorted.heterozygous.vcf

# use GATK to make a new reference for each haplotype using 1) the original vcf 2) the homozygous vcf only

java -jar GenomeAnalysisTK.jar -R GRCh37-lite.fa -T FastaAlternateReferenceMaker -o alt_ref_hap1.fa --variant Illumina_body_map/HCT20170/ERS025093_5_lanes_dupsFlagged.sorted.vcf

java -jar GenomeAnalysisTK.jar -R GRCh37-lite.fa -T FastaAlternateReferenceMaker -o alt_ref_hap2.fa --variant Illumina_body_map/HCT20170/ERS025093_5_lanes_dupsFlagged.sorted.homozygous.vcf

# munge the header lines generated in the GATK back into the correct fasta header lines (from the original reference)

gawk 'FNR==NR{/^>/ ? m=1 : m =0 ; if (m==1){b++; a[b]=$1;next} else {next}} { /^>/ ? m=1 : m=0 ; if (m==1) {c++; print a[c]} else {print $0}}' GRCh37-lite.fa alt_ref_hap1.fa > alt_ref_hap1.with_headers.fa

rm alt_ref_hap1.fa

gawk 'FNR==NR{/^>/ ? m=1 : m =0 ; if (m==1){b++; a[b]=$1;next} else {next}} { /^>/ ? m=1 : m=0 ; if (m==1) {c++; print a[c]} else {print $0}}' GRCh37-lite.fa alt_ref_hap2.fa > alt_ref_hap2.with_headers.fa

rm alt_ref_hap2.fa

# split the new reference into files (one chromosome per file) and add "chr" to each of the filenames

cd /folder/to/contain/reference1

awk '/^>/ {OUT=substr($1,2) ".fa"}; OUT {print >OUT}' alt_ref_hap1.with_headers.fa

for i in *; do mv "$i" `echo "$i" | sed 's/^/chr/'`; done

cd /folder/to/contain/reference2

awk '/^>/ {OUT=substr($1,2) ".fa"}; OUT {print >OUT}' alt_ref_hap2.with_headers.fa

for i in *; do mv "$i" `echo "$i" | sed 's/^/chr/'`; done

# run flux simulator on first haplotype and hap1.par

flux-simulator -t simulator -x -l -s -p hap1.par

# run flux simulator on second haplotype reference using only -s and hap2. par

flux-simulator -s -p hap2.par

#rename the second haplotype to avoid having 2 fragments with the same read name

cat hap2.fastq | awk '{if ($0~/^@/) {gsub(/^@/,"@:hap2:"); print} else {print $0}}' > hap2.renamed.fastq

#merge the fastqs for the two haplotypes

cd /projects/mkreitzman_prj/RNA-seq_simulation/Flux_simulator

cat hap1.fastq hap2.renamed.fastq > all.fastq

#separate merged fastq into 1st and 2nd end files.

perl split_interleaved_fastq.pl

FluxSimulator v1.2.1 was run with the par files:

Hap1.par:

### input, output ###

NB_MOLECULES 5000000

REF_FILE_NAME ensembl_fromUCSC_no_scaff_sorted.gtf

GEN_DIR RNA-seq_simulation/GATK/alt_ref_hap1_split_ref

### Expression ###

TSS_MEAN 50

POLYA_SCALE NaN

POLYA_SHAPE NaN

### Fragmentation ###

FRAG_SUBSTRATE RNA

FRAG_METHOD UR

FRAG_UR_ETA 250

FRAG_UR_D0 1

### Reverse Transcription ###

RTRANSCRIPTION YES

RT_PRIMER RH

RT_LOSSLESS YES

RT_MIN 500

RT_MAX 5500

### Amplification ###

GC_MEAN NaN

PCR_PROBABILITY 0.05

FILTERING NO

### Sequencing ###

READ_NUMBER 100000000

READ_LENGTH 100

PAIRED_END YES

ERR_FILE 76

FASTA YES

UNIQUE_IDS YES

Hap2. Par :

### input, output ###

NB_MOLECULES 5000000

REF_FILE_NAME ensembl_fromUCSC_no_scaff_sorted.gtf

GEN_DIR RNA-seq_simulation/GATK/alt_ref_hap2_split_ref

PRO_FILE_NAME RNA-seq_simulation/Flux_simulator/hap1.pro

LIB_FILE_NAME RNA-seq_simulation/Flux_simulator/hap1.lib

### Expression ###

TSS_MEAN 50

POLYA_SCALE NaN

POLYA_SHAPE NaN

### Fragmentation ###

FRAG_SUBSTRATE RNA

FRAG_METHOD UR

FRAG_UR_ETA 250

FRAG_UR_D0 1

### Reverse Transcription ###

RTRANSCRIPTION YES

RT_PRIMER RH

RT_LOSSLESS YES

RT_MIN 500

RT_MAX 5500

### Amplification ###

GC_MEAN NaN

PCR_PROBABILITY 0.05

FILTERING NO

### Sequencing ###

READ_NUMBER 100000000

READ_LENGTH 100

PAIRED_END YES

ERR_FILE 76

FASTA YES

UNIQUE_IDS YES
